# HLA-DR modulation and PD-1/PD-L2 checkpoint signaling identify a mechanistic potency axis for mesenchymal stromal cell immunosuppression

**DOI:** 10.1093/stcltm/szag090

**Published:** 2026-11-05

**Authors:** Mahin Nikougoftar Zarif, Katia Lefsihane, Neda Khanlarkhani, Lina Sorvik, Jan Fredrik Talts, Katarina Le Blanc, Nadir Kadri

**Affiliations:** Research and Development Department, Cellcolabs AB, Solna, Stockholm, 17165, Sweden; Research and Development Department, Cellcolabs AB, Solna, Stockholm, 17165, Sweden; Research and Development Department, Cellcolabs AB, Solna, Stockholm, 17165, Sweden; Research and Development Department, Cellcolabs AB, Solna, Stockholm, 17165, Sweden; Research and Development Department, Cellcolabs AB, Solna, Stockholm, 17165, Sweden; Department of Laboratory Medicine, Karolinska Institute, Huddinge, Stockholm, 14186, Sweden; Department of Laboratory Medicine, Karolinska Institute, Huddinge, Stockholm, 14186, Sweden

**Keywords:** mesenchymal stromal cells, HLA-DR, PD-1, potency assay, mixed lymphocyte reaction

## Abstract

**Introduction:**

Mesenchymal stromal cells exhibit potent immunomodulatory properties and are under active investigation for the treatment of immune-mediated disorders. However, their clinical translation is hindered by the lack of standardized potency assays.

**Methods:**

Here, we established a reproducible mixed lymphocyte reaction platform by systematically optimizing peripheral blood mononuclear cell donor composition, culture conditions, and co-culture ratios to define a robust activation window.

Using this optimized mixed lymphocyte reaction-based potency assay, we compared bone marrow and adipose-derived mesenchymal stromal cells across independent donor batches.

**Results:**

Both sources of mesenchymal stromal cells effectively suppressed T-cell proliferation, with the adipose-derived source demonstrating greater inhibitory activity under the experimental conditions, while a conserved lower threshold of suppression was observed across both sources. Mesenchymal stromal cells reduced early (CD25^+^) and late (CD25^+^ HLA-DR^+^) T-cell activation, with downregulation of CD25 and HLA-DR emerging as sensitive correlates of functional potency. Notably, bone marrow-derived mesenchymal stromal cells exerted stronger suppression of late-stage HLA-DR^+^ activation and preferentially inhibited CD8^+^ T-cell expansion, demonstrating source-dependent immunomodulatory functions. Mechanistically, this suppression was associated with modulation of the PD-1 pathway, characterized by decreased soluble PD-1, increased soluble PD-L1, and induction of soluble PD-L2 by mesenchymal stromal cells. PD-L2 levels inversely correlated with T-cell proliferation in the mixed lymphocyte reaction, supporting a PD-1/PD-L2 regulatory axis linked to mesenchymal stromal cell potency.

**Conclusion:**

These findings support a standardized and mechanistically informed mixed lymphocyte reaction-based potency assay framework integrating functional suppression, HLA-DR modulation, and PD-1/PD-L2 pathway signaling as complementary indicators of mesenchymal stromal cell activity.

## Graphical Abstract

**Graphical Abstract:**
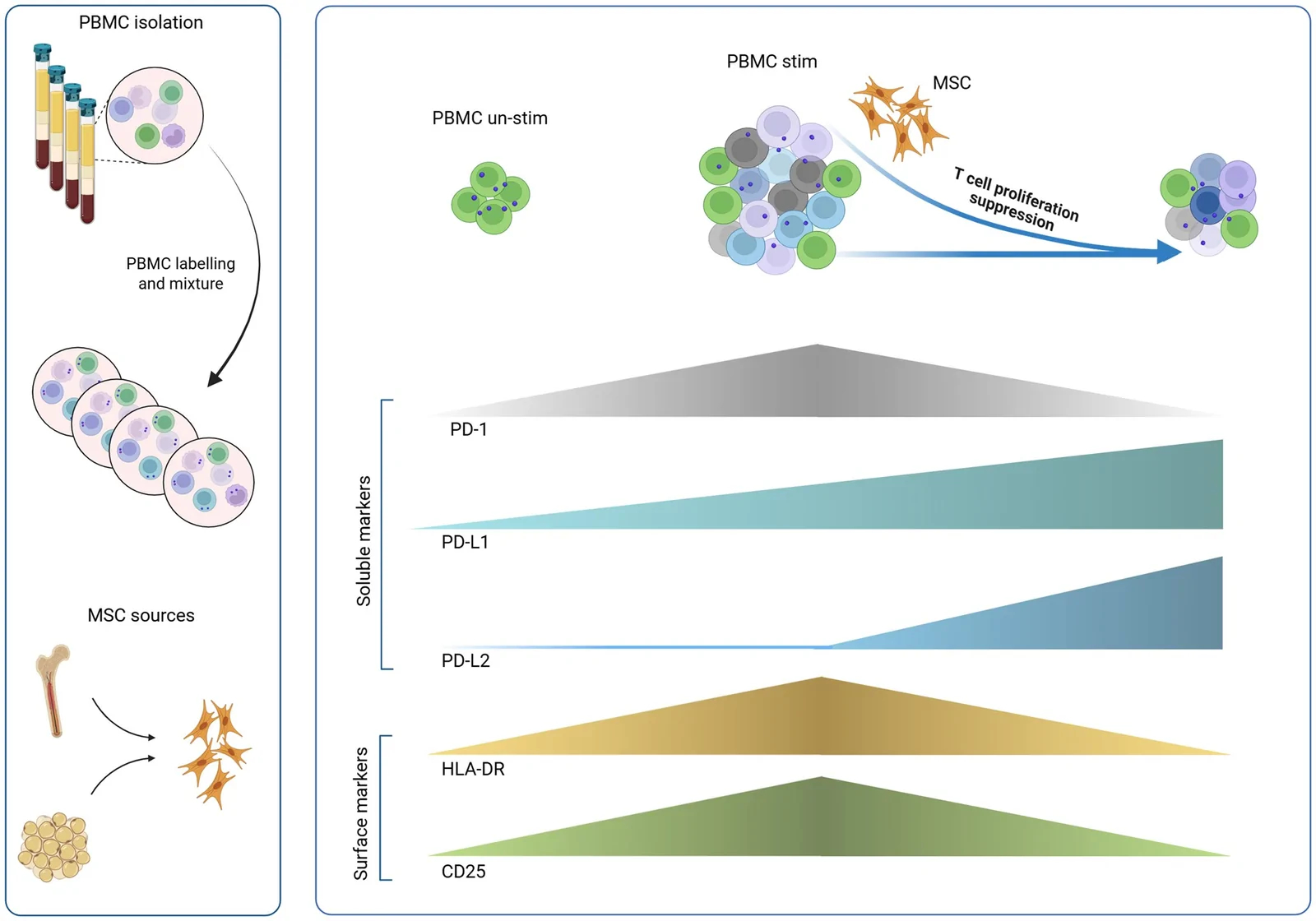

Significance statement The clinical translation of mesenchymal stromal cells (MSCs) is limited by the lack of standardized potency assays. We developed a reproducible mixed lymphocyte reaction-based assay by optimizing donor composition, culture conditions, and MSC-to-peripheral blood mononuclear cells ratios. Adipose- and bone marrow-derived MSCs showed distinct immunomodulatory profiles, while HLA-DR downregulation and preferential CD8^+^ T-cell suppression emerged as sensitive functional readouts. MSC-mediated suppression was also associated with PD-1/PD-L2 pathway modulation, highlighting PD-L2 as a potential potency marker. This framework supports quantitative and mechanistically informed MSC potency assessment.

## Introduction

Mesenchymal stromal cells (MSCs) are multipotent progenitor cells present in multiple tissues and contribute to tissue repair and immune regulation. They are defined by the International Society for Cellular Therapy (ISCT) through plastic adherence, tri-lineage differentiation capacity, and characteristic surface marker expression.^1^ MSCs exhibit potent immunomodulatory properties through the secretion of soluble factors and extracellular vesicles, making them promising candidates for treating immune-mediated diseases.^2^^,^^3^

MSCs regulate immune responses by suppressing T-cell activation and proliferation, modulating antigen-presenting cells and promoting regulatory T-cell induction.^2^^,^^4^ Both CD4^+^ and CD8^+^ T-cell subsets are affected, with activation marked by early CD25 expression and later HLA-DR upregulation. Modulation of these activation states may provide informative functional readouts beyond proliferation-based assays.^5–7^

Despite clinical success in indications such as graft versus host disease (GVHD),^8^ a major challenge in the clinical development of MSCs-based therapies is the lack of standardized potency assays. Regulatory agencies emphasize the need for robust, quantitative assays that reflect the mechanism of action relevant to the intended clinical indication.^9^ The mixed lymphocyte reaction (MLR) is widely used to measure MSCs-mediated immunosuppression,^10^ but is highly sensitive to experimental variability, underscoring the need for optimized and reproducible assay platforms suitable for quality control.

Additionally, MSCs from different tissue sources exhibit functional heterogeneity, including differences in immunosuppressive capacity, molecular profiles, and variation in surface molecules such as CD142, also known as tissue factor (TF), which may influence their function. These variations highlight the need for standardized systems to enable meaningful comparison between MSCs products.^11^^,^^12^

Immune checkpoint pathways, particularly programmed death (PD)-1 signaling, have emerged as key regulators of MSCs and T-cell interactions.^13^ PD-1 engagement by its ligands programmed death-ligand (PD-L)1 and PD-L2 attenuates T-cell activation and functions; however, the relationship between checkpoint modulation and quantitative potency readouts remains unclear.^14^^,^^15^

In this study, we established a standardized MLR platform by optimizing key experimental variables to define a reproducible activation window. Using this system, we compared bone marrow (BM)- and adipose (AD)-derived MSCs across donor batches, evaluated T-cell activation dynamics, and identified modulation of late activation states as a sensitive functional correlate. We further demonstrated that MSC-mediated immunosuppression is associated with modulation of a PD-1/PD-L2 regulatory axis linked to functional potency, providing a rationale for further evaluation of this pathway as a potential component of future MSC potency assays.

## Methods

### Peripheral blood mononuclear cells preparation

Six buffy coats from healthy donors were obtained from Karolinska University Hospital with informed consent and under ethical approval from the Swedish Ethical Review Authority (Ethicprövningsmyndigheter) reference number 2024-01747-01. PBMCs were isolated using Ficoll-Paque™ PLUS (Cytiva, Sweden), cryopreserved in CryoStor® CS10 (StemCell Technologies), and stored in liquid nitrogen until use. For each experiment one cryovial from each donor was thawed, washed with RPMI 1640 (Gibco, USA) supplemented with 10% USDA-approved fetal bovine serum (FBS; Gibco, USA) to constitute the complete medium, and labeled with the Cell Trace™ Violet (CTV) Cell Proliferation Kit (Thermofisher, USA) according to the manufacturer’s instructions. The labeled PBMCs were then resuspended at a final concentration of 2 × 10^6^ cells/mL in complete medium. From the six individual donors, 15 distinct four donor-PBMC mixtures were prepared by combining equal volumes of cells from unrelated donors to induce an allogeneic lymphocyte reaction. Owing to human leukocyte antigen (HLA) mismatches between donors, alloreactive T-cells became activated and proliferated, allowing assessment of variability in T-cell proliferation among different donor combinations. In parallel, a six donor PBMC mixture was prepared to evaluate whether increasing donor diversity influenced the proliferative response of T-cells (Supplementary Table A). Unstimulated controls consisted of PBMCs cultured from individual donors without allogeneic mixing.

To assess the impact of culture supplements on T-cell proliferation, PBMC mixtures were cultured in RPMI 1640 media supplemented with (i) USDA-approved FBS, (ii) research use only (RUO) FBS (Gibco, USA), or (iii) human platelet lysate (HPL) (PL Bioscience GmbH, Germany). Since MSCs were expanded in medium supplemented with 5% HPL, this condition was included for comparison. For co-culture experiments, MSCs at passage three were used. A total of 2 × 10^5^ mixed PBMCs were seeded per well in 96-well V-bottom plates.

### MSCs preparation

MSCs were isolated from healthy donors following informed consent and under ethical approval from the Swedish Ethical Review Authority (Ethicprövningsmyndigheter) reference number 2025-08474-01, including four bone marrow and four adipose tissue samples. Mononuclear cells (MNCs) were isolated by density gradient centrifugation, and BM-derived cells were cultured in Dulbecco’s Modified Eagle Medium (DMEM; Gibco, USA) while AD-derived cells were cultured in Minimum Essential Medium Alpha (MEM-α; Gibco, USA). Both media were supplemented with 5% HPL. Cells were maintained under standard culture conditions and passaged upon reaching approximately 80% confluence. MSCs were characterized according to ISCT criteria based on our previous article.^1^ All cell preparations were cryopreserved at passage three. The number of samples and production batches are summarized in Supplementary Tables B and C.

### Mixed lymphocyte reaction

Cryopreserved MSCs from passage three were used for all experiments. AD-derived MSCs from four donors (eight batches) and BM-derived MSCs from four donors (nine batches) were thawed and washed in RPMI 1640 supplemented with 10% USDA-approved FBS. Cells were resuspended at a concentration of 5 × 10^5^ cells/mL prior to co-culture.

MSCs were co-cultured with PBMC mixtures at MSCs: PBMC ratios of 1:4, 1:8, and 1:16 in triplicate in 96-well V-bottom plates, with a final medium volume of 200 µL per well. Cultures were maintained for seven days. Unstimulated PBMCs and stimulated PBMCs without MSCs were cultured as negative and positive controls, respectively. On day three, partial medium exchange was performed by gently centrifuging the plate (3 min at 300 g) to sediment the cells, removing 80 µL of culture supernatant and replacing it with 100 µL of fresh complete medium. On day seven, the supernatants were collected for ELISA, and cells were stained for analysis by FACS (Figure 1A).

**Figure 1.**
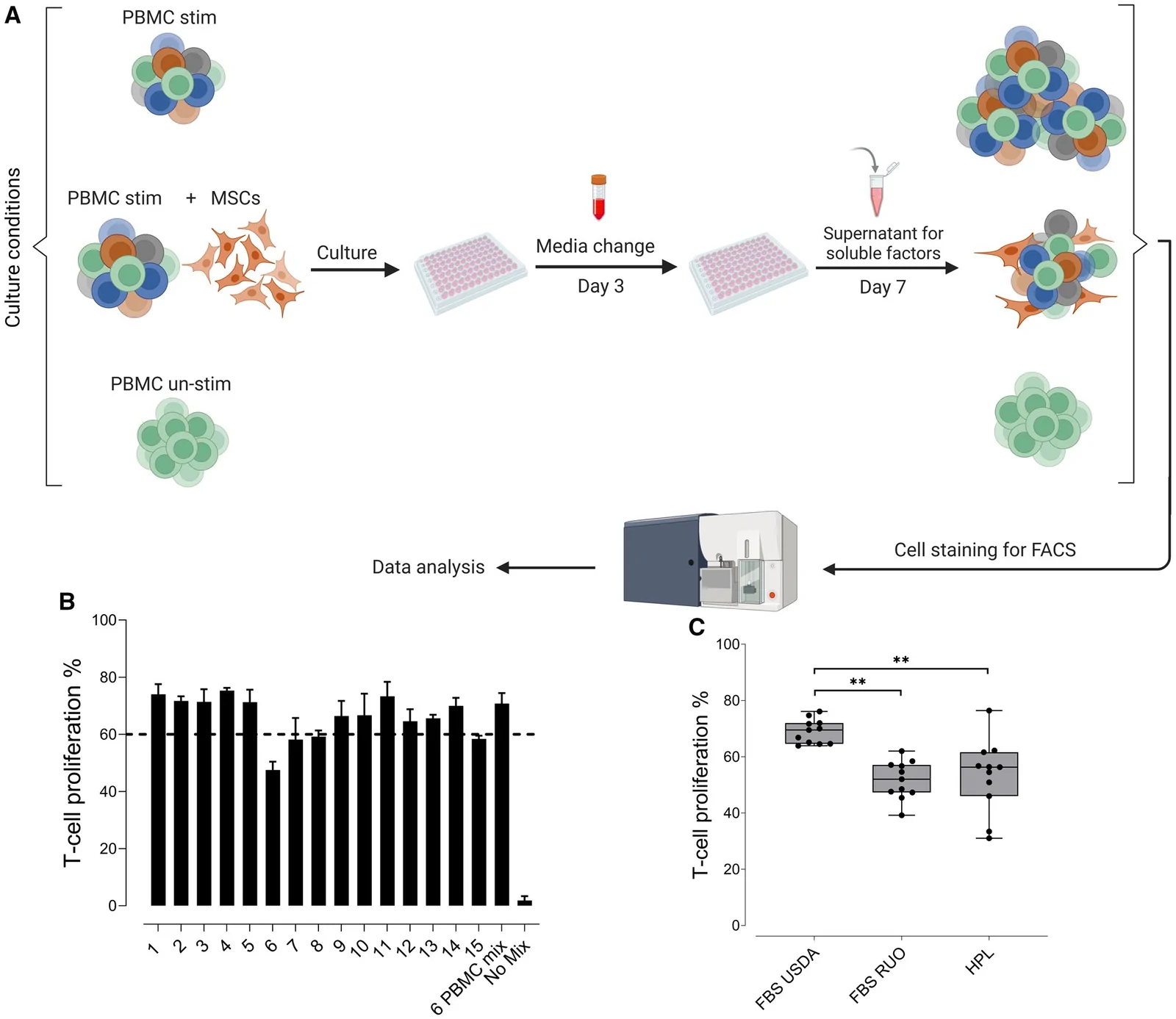
PBMC and MSCs co-culture workflow and optimization of the mixed lymphocyte reaction assay. (A) Schematic overview of the PBMC and MSCs co-culture assay. (B) T-cell proliferation (%) in 15 different combinations of PBMC from four donors and one combined mixture of six donors. Data is presented as mean ± SD from three technical replicates. The dashed horizontal line indicates the 60% proliferation threshold used for selection of the PBMC donor mixture. (C) T-cell proliferation (%) in selected PBMC mixtures cultured with different medium supplements. Data are presented as mean ± SD from 11 PBMC donor combinations, with each combination analyzed in technical replicates. Statistical significance was determined by repeated-measures one-way ANOVA followed by Sidak’s multiple comparisons test (**P ≤ .01). FBS, fetal bovine serum; HPL, human platelet lysate; MSC, mesenchymal stromal cells; PBMC, peripheral blood mononuclear cells; SD, standard deviation.

### Flow cytometry analysis

After seven days of culture, cells were stained with PE- or FITC-conjugated anti-human CD3 (both from eBioscience, USA) and Fixable Viability Dye eFluor™ 780 (Invitrogen, USA). Cells were subsequently fixed using Cytofix/Cytoperm solution (BD Biosciences, USA). T-cell proliferation was assessed based on CTV dilution within single viable CD3^+^ T-cells (Figure S1A and B). Samples were run in a CytoFLEX flow cytometer, and data were analyzed with FlowJo software (v10.0).

To minimize inter-experimental variability, T-cell proliferation was normalized to the proliferation rate observed in the PBMC mixture control within each independent experiment. The suppressive effect of MSCs was calculated as the percentage of Normalized T-cell Proliferation Reduction (%NTPR) using the following formula:


$$\%N\mathrm{TPR}=100-\left(\frac{TC}{TS}\times 100\right)$$

where *TC* represents the percentage of proliferating T-cells in co-culture with MSCs, and *TS* represents the percentage of proliferating T-cells in the stimulated PBMC mixture control.

### CD142 (TF) and Podoplanin detection

The surface expression of CD142 and Podoplanin on CD73+ MSCs was assessed by flow cytometry. Cells were stained with FITC-conjugated anti-human CD73 antibody, PE-conjugated anti-human CD142 antibody, and APC-conjugated anti-human Podoplanin (all from eBioscience, USA). Isotype controls and unstained cells were used to exclude nonspecific binding and background fluorescence, respectively.

### Detection of CD4, CD8, CD25, and HLA-DR in MLR

To evaluate MSCs-mediated suppression of T-cell activation and proliferation, surface expression of CD25, HLA-DR together with CD4 and CD8 was analyzed within single live T-cells (using FITC-conjugated anti-human CD3) following the MLR. Cells were stained using PE-conjugated anti-human CD25 (Biolegend, USA), PerCP-Cy5-conjugated anti-human HLA-DR (Beckton Dickinson, USA), APC-conjugated anti-human CD4 (Biolegend, USA), and BV605-conjugated anti-human CD8 (Biolegend, USA) according to standard staining protocols.

### Soluble PD-1, PD-L1, and PD-L2 quantification

Supernatants were collected from each MLR condition on day seven and stored at −80 °C until analysis. Soluble PD-1, PD-L1, and PD-L2 concentrations were quantified using commercially available ELISA kits (Abcam, Cambridge, UK) according to the manufacturer’s instructions. Optical density was measured at 450 nm using a microplate ELISA reader (PerkinElmer, USA), and concentrations were calculated from standard curves generated in parallel with each assay. To account for inter-experimental variability, PD-1 and PD-L1 concentrations in MSCs and PBMC co-cultures were normalized to stimulate PBMC control. Normalized concentrations were calculated using the following formula:


Normalized concentration (%)=(CC×100)/SC

where *CC* represents the concentration of the target molecule in MSCs co-cultured with stimulated PBMCs, and *SC* represents the concentration of the same molecule in the stimulated PBMC condition alone. In contrast, PD-L2 levels were reported as absolute concentrations, as PD-L2 was not detectable in the stimulated PBMC condition.

### Statistical analysis

Statistical analyses were performed using GraphPad Prism (version 10; GraphPad Software, San Diego, CA, USA). For experiments performed in technical triplicates, the mean of the three technical replicates was used as a single data point for statistical analysis. NTPR data were analyzed at donor level to avoid pseudo-replication. For comparisons between AD- and BM-derived MSCs, donor level means were treated as independent biological replicates, whereas multiple batches originating from the same donor were considered repeated measurements and were not treated as independent biological replicates. Comparisons involving multiple matched experimental conditions were analyzed using one-way ANOVA followed by the appropriate post hoc multiple comparisons test as specified in the figure legends. Comparisons between two independent groups were performed using an unpaired two-tailed Student’s *t*-test. Correlations were assessed using Spearman’s rank correlation analysis. A *P* value < .05 was considered statistically significant.

## Results

### Standardization of an MLR assay identifies key biological determinants influencing MSCs-mediated immunosuppression

To establish a robust platform for assessing the immunomodulatory potency of MSCs, we first optimized an MLR-based assay modeling allogeneic immune responses (Figure 1A). T-cell proliferation was evaluated across multiple combinations of PBMCs derived from independent donors to define a suitable activation window (Supplementary Table A). Among the 15 donor combinations tested, eleven exhibited proliferations exceeding 60%, with a variation of less than 13% between them, indicating a reproducible activation window within this range. In contrast, four donor combinations generated lower proliferative responses ranging between 49% and 60%, reflecting weaker allogeneic stimulation (Figure 1B). Based on these optimization experiments, donor combinations with >60% T-cell proliferation were selected for subsequent assays because they provided a robust and reproducible allogeneic response with sufficient dynamic range to detect differences in MSC-mediated immunosuppression. This cutoff was chosen empirically to standardize the assay rather than as a disease-specific threshold. Previous studies have also shown that strongly alloreactive MLRs are characterized by high levels of T-cell proliferation, supporting the use of highly responsive donor combinations for potency testing.^16^ We also found that this activation threshold can be affected by nutrients into the assay, as adding another type of FBS or HPL significantly reduced in the proliferation of PBMCs (Figure 1C). Furthermore, MSCs were co-cultured with PBMCs in three different ratios. Among these, the ratio of 1:4 in MSC: PBMC demonstrated the greatest suppressive effect and was therefore selected for all subsequent experiments (Figure S1C). These findings demonstrate that MLR-based potency assays are highly sensitive to donor composition and culture conditions highlighting the importance of assay standardization when comparing MSCs products derived from different tissue sources or manufacturing runs.

### Quantitative potency thresholds reveal enhanced immunosuppressive activity of adipose-derived MSCs in the mixed lymphocyte reaction

Using the optimized MLR system, MSCs derived from BM and AD tissues were evaluated for their ability to suppress T-cell proliferation in stimulated PBMC cultures. Both MSC sources exhibited robust immunosuppressive activity. To illustrate the variability among MSC preparations, the NTPR values of all individual samples are shown in Figure 2A. These data are presented descriptively only and therefore were not treated as independent biological replicates. For donor-level analysis, the mean NTPR value for each donor was used as the independent biological replicate. Based on this analysis, AD-derived MSCs demonstrated significantly greater inhibition of T-cell proliferation than BM-derived MSCs (Figure 2B). Nevertheless, across all experimental replicates, the lowest suppression observed for AD-derived MSCs was 40%, whereas BM-derived MSCs displayed a minimal suppression of 34%. Importantly, when normalized to the median inhibitory response within the assay, these values corresponded to similar proportional reduction of approximately 66.8% and 67.7%, respectively. This convergence across independent experiments suggests the presence of a reproducible lower functional boundary of MSC-mediated immunosuppression regardless of the cell origin within this MLR system. Taken together, these observations provide a practical reference range for benchmarking of MSC potency evaluation, while preserving sufficient assay sensitivity to resolve biologically meaningful differences in immunosuppressive capacity among MSC preparations (Figure 2C and D).

**Figure 2.**
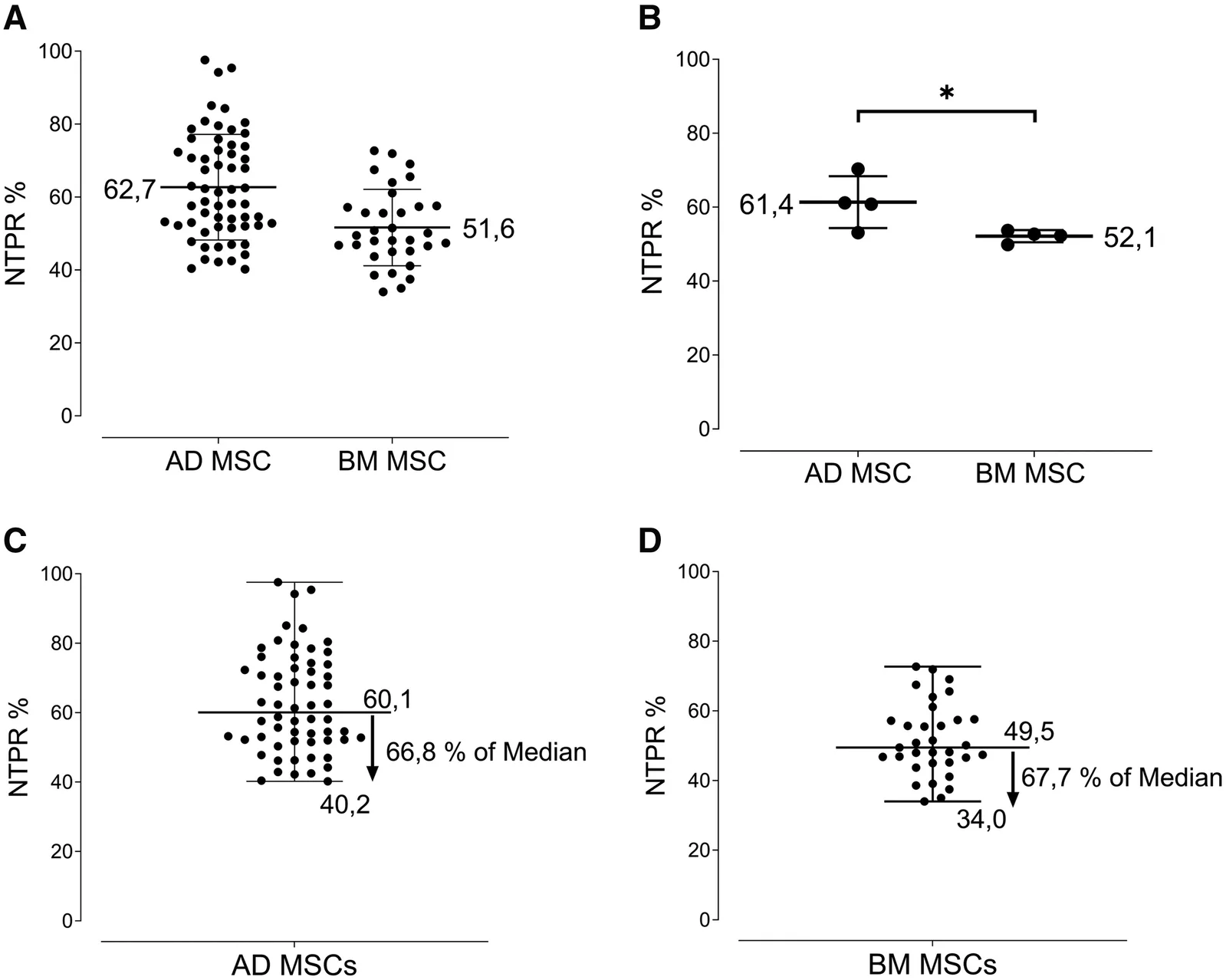
Immunomodulatory potency of adipose and bone marrow-derived MSCs. (A) Distribution of NTPR values for all individual MSC samples. These data are presented to illustrate variability among MSC preparations and are intended for descriptive purposes only; batch-level measurements were not treated as independent biological replicates in statistical analyses. (B) NTPR (%) in stimulated PBMCs co-cultured with AD- and BM-derived MSCs. Data was analyzed at the donor-level from eight biological replicates (four AD- and four BM-derived MSC donor preparations), with each donor value representing the mean of all available MSC batches from that donor. Data are presented as mean ± SD. Statistical significance was determined using an unpaired two-tailed Student’s *t*-test (*P ≤ .05). Each biological replicate was analyzed in technical triplicate, and the mean of the technical replicates was used for statistical analysis. (B). (C and D) Median NTPR and observed range for AD (C)- and BM (D)-derived MSCs. The upper horizontal line indicates the maximum NTPR, whereas the lower horizontal line represents the minimum observed suppressive activity. The calculated lower functional boundaries corresponded to 66.8% and 67.7% of the median NTPR for AD- and BM-derived MSCs, respectively. Each measurement was performed in technical triplicate, with the mean of the technical replicates used for analysis. AD, adipose; BM, bone marrow; MSC, mesenchymal stromal cells; NTPR, normalized T-cell proliferation reduction; PBMC, peripheral blood mononuclear cells; SD, standard deviation.

Phenotypic characterization revealed that AD- and BM-derived MSCs displayed comparable expression of canonical MSCs markers included in the ISCT release criteria, including CD90, CD73, and CD105, and lacking expression of hematopoietic and endothelial markers such as CD34 and CD31. In contrast, differential expressions emerged for tissue factor CD142 and the motility marker Podoplanin (Figure 3A). Both markers displayed substantial heterogeneity across individual MSC samples, indicating donor-dependent variability in the expression of functional stromal markers (Figure 3B and C). These findings suggest that while both MSCs populations conform to established identity criteria, source-associated variation in functional and stromal markers may be associated with differences in immunomodulatory activity.

**Figure 3.**
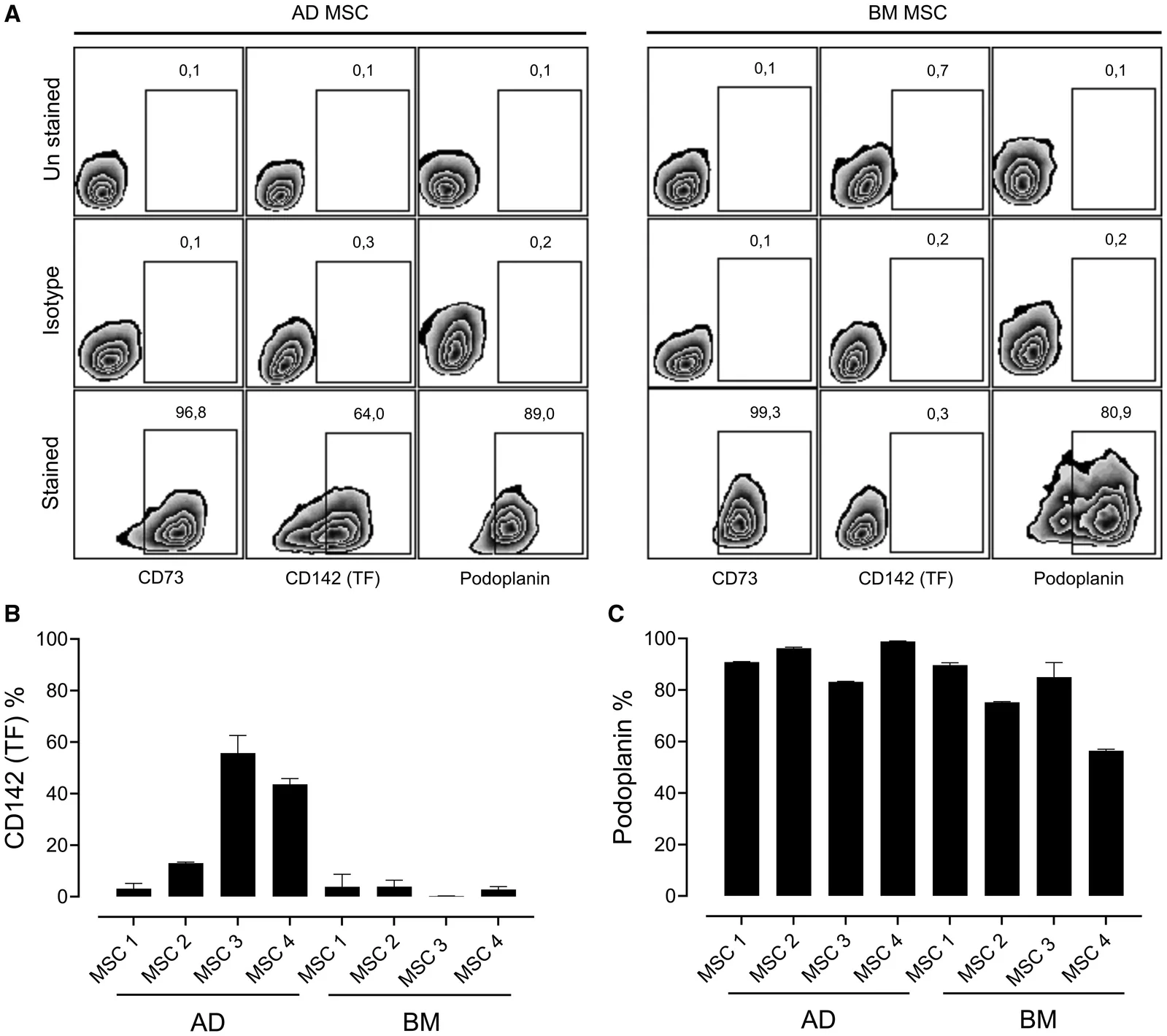
Expression of CD142 and Podoplanin in AD- and BM-derived MSCs. (A) Representative flow cytometry plots, showing the expression of CD142 (tissue factor, TF) and Podoplanin on CD73+ MSCs. Unstained and isotype controls are shown for comparison. Numbers indicate the percentage of positive cells within the gated MSC population. (B and C) Percentage of CD142 (TF) positive (B) and Podoplanin positive (C) MSCs from eight biological replicates (four AD- and four BM-derived MSC donor preparations). Considerable donor-to-donor variability in CD142 expression was observed, particularly among AD MSCs, whereas Podoplanin expression was consistently high in both MSC sources, with modest inter-donor variation. Data are presented as mean ± SD for eight different samples which analyzed in technical triplicate and the mean of the technical replicates used for analysis. AD, adipose; BM, bone marrow; MSC, mesenchymal stromal cells; SD, standard deviation.

### HLA-DR downregulation on activated T-cells serves as a novel sensitive surrogate marker of MSCs functional activity

To further delineate the immunological mechanisms underlying MSC-mediated suppression within this system, we examined the expression of T-cell activation markers following co-culture with MSCs. The early activation marker CD25 and the late activation marker HLA-DR were assessed to capture distinct stages of T-cell activation dynamics. To our knowledge, modulation of HLA-DR expression on activated T-cells following MSCs exposure received limited attention as a functional readout.

In mixed lymphocyte reactions, CD25 expression was first induced following PBMC activation and was subsequently accompanied by a progressive increase in HLA-DR expression within the responding T-cell population (Figure 4A). Across independent experiments, MSC co-culture reduced both CD25+ and CD25+ HLA-DR+ T-cell populations, consistent with the observed suppression of proliferation (Figure 4B). Notably, the reduction in HLA-DR expression was significantly greater in co-cultures containing BM-derived MSCs compared with AD-derived MSCs, suggesting that MSCs from BM exert a stronger regulatory influence on late-stage T-cell activation during allogeneic immune responses (Figure 4C).

**Figure 4.**
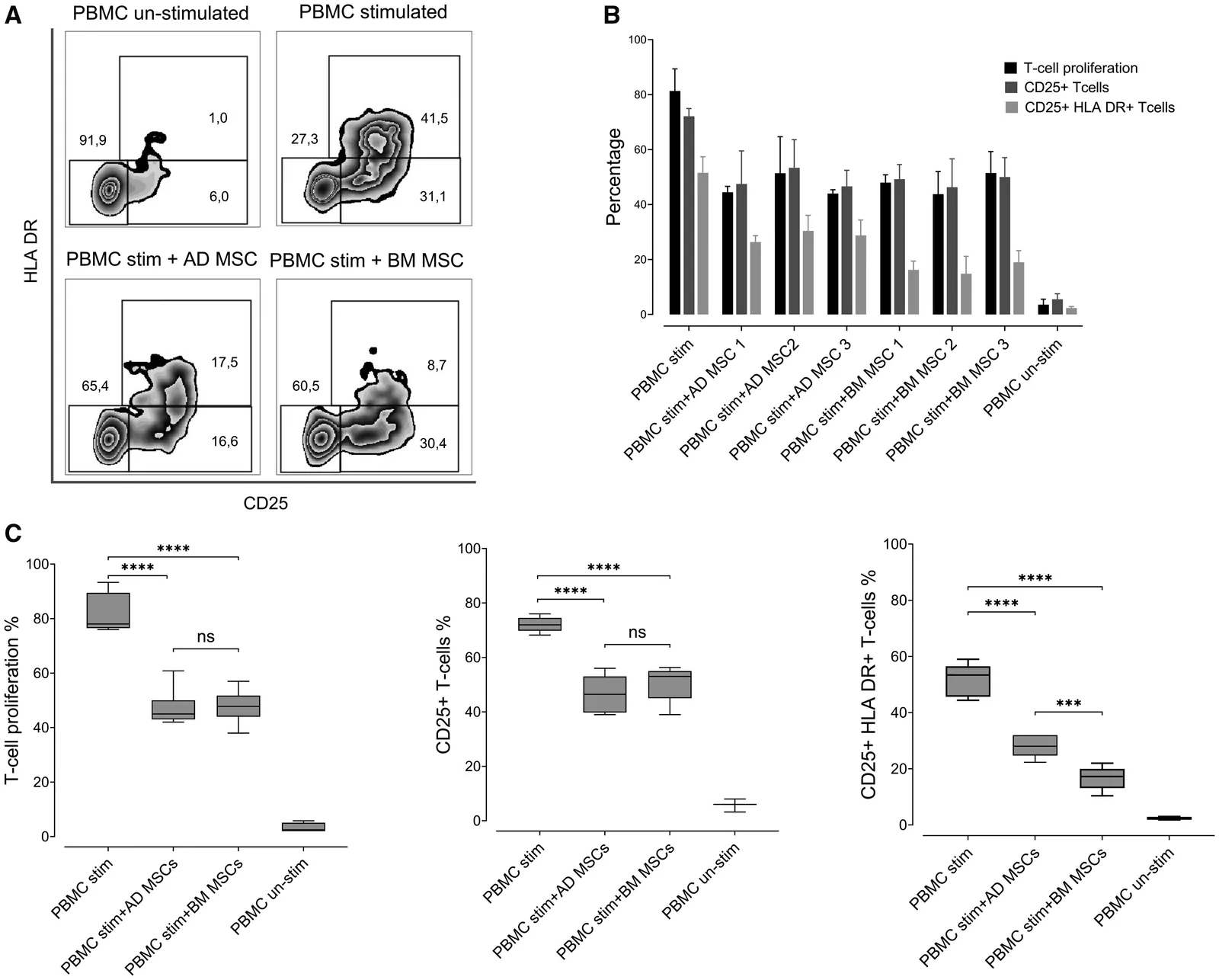
Expression of CD25 and HLA-DR on T-cells under MLR conditions. (A) Representative flow cytometry plots showing CD25 and HLA-DR expression on T-cells under different conditions: stimulated PBMCs, unstimulated PBMCs, co-culture with AD MSCs, and co-culture with BM-derived MSCs. (B) Percentage of proliferating T-cells (based on CTV dilution), CD25^+^ T-cells, and CD25^+^HLA-DR^+^ T-cells under the different MLR conditions. Each bar represents four different experiments from the same donor. Each technical triplicates within the experiments were averaged before analysing. (C) Comparison of T-cell proliferation, CD25^+^ T-cells, and CD25^+^HLA-DR^+^ T-cells in different conditions. Statistical significance was determined using one-way ANOVA followed by Tukey’s test. (***P ≤ .001, ****P ≤ .0001). Data in C are presented as mean ± SD from four different experiments, including two sets of three biological replicates (three AD- and three BM-derived MSC donor preparations). Each biological replicate analyzed in technical triplicate, and the mean of the technical replicates used for analysis. AD, adipose; BM, bone marrow; CTV, CellTrace Violet; MSC, mesenchymal stromal cells; MLR, mixed lymphocyte reaction; PBMC, peripheral blood mononuclear cells; SD, standard deviation.

To define the impact of MSCs-mediated suppression on T-cell subsets, we analyzed CD4^+^ and CD8^+^ T-cell populations within the MLR system. Both subsets showed robust proliferation during allogeneic stimulation (Figure 5A). Also, our data showed that in the presence of MSCs, a differential suppressive effect emerged, with a more pronounced reduction within the CD8^+^ T-cell compartment. This shift was reflected in the CD4/CD8 ratio, where co-culture conditions more closely resembled the baseline ratio observed in unstimulated PBMCs (Figure 5B and C), suggesting preferential suppression of CD8^+^ T-cell expansion.

**Figure 5.**
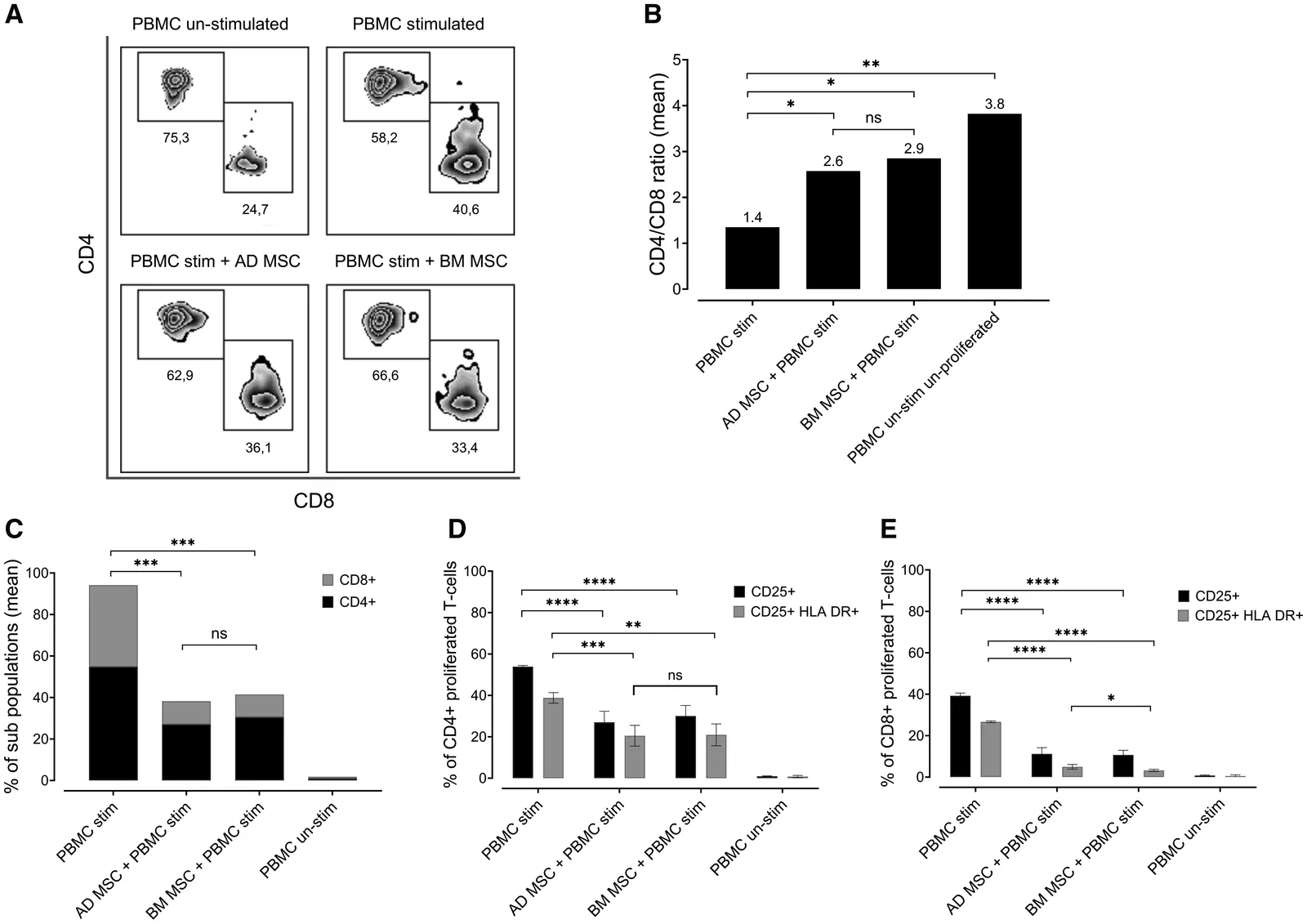
Analysis of T-cell subpopulations under MLR conditions. (A) Representative flow cytometry plots showing the distribution of proliferated CD4^+^ and CD8^+^ T-cells subpopulations in unstimulated PBMCs, stimulated PBMCs, and stimulated PBMCs co-cultured with AD- and BM-derived MSCs. (B) The mean of CD4^+^/CD8^+^ T-cells ratio across different MLR conditions. (C) Distribution of proliferated CD4^+^ and CD8^+^ T-cell subsets (mean) under the different MLR conditions. (D) Percentage of CD25^+^ and CD25^+^HLA-DR^+^ T-cells within the proliferated CD4^+^ T-cell population (mean ± SD). (E) Percentage of CD25^+^ and CD25^+^ HLA-DR^+^ T-cells within the proliferated CD8^+^ T-cell population (mean ± SD). Data are presented from four different experiments, including two sets of four biological replicates (four AD- and four BM-derived MSC donor preparations), with each biological replicate analyzed in technical triplicate and the mean of the technical replicates used for analysis. Statistical significance was determined using repeated measures one-way ANOVA followed by Tukey’s test (*P ≤ .05, **P ≤ .01, ***P ≤ .001, ****P ≤ .0001). AD, adipose; BM, bone marrow; MSC, mesenchymal stromal cells; MLR, mixed lymphocyte reaction; PBMC, peripheral blood mononuclear cells; SD, standard deviation.

We further assessed activation markers within each subset by quantifying CD25+ and CD25^+^ HLA-DR^+^ populations. MSCs resulted in a marked reduction of both activated populations of CD4^+^ and CD8^+^ T-cells, confirming effective suppression of activation in both lineages (Figure 5D and E). Notably, the decrease in the late activated CD25^+^ HLA-DR^+^ population was more pronounced in co-cultures containing BM-derived MSCs compared with AD-derived MSCs, indicating a stronger inhibitory effect of BM-derived MSCs on late-stage T-cell activation (Figure 5E).

Collectively, these findings identify downregulation of HLA-DR as a functional correlate of MSCs-mediated immunosuppression and support the concept that monitoring late activation states of T-cells may provide a sensitive and mechanistically informative surrogate parameter for MSCs potency assessment in MLR-based assays.

### MSCs-mediated immunosuppression is associated with activation of a PD-1/PD-L2 regulatory axis

To explore soluble mediators contributing to MSCs-induced immunoregulation in MLR, we next examined components of the PD-1 immune checkpoint pathway. Supernatant from MSCs co-culture experiments were collected at day seven of coculture. Soluble PD-1, PD-L1, and PD-L2 were quantified in coculture supernatants by ELISA.

Activated PBMC cultures without MSCs produce detectable levels of soluble PD-1, consistent with T-cell activation. However, the addition of MSCs isolated from both BM and AD tissue resulted in a significant reduction in soluble PD-1 levels, paralleling the observed suppression of T-cell proliferation (Figure 6A).

**Figure 6.**
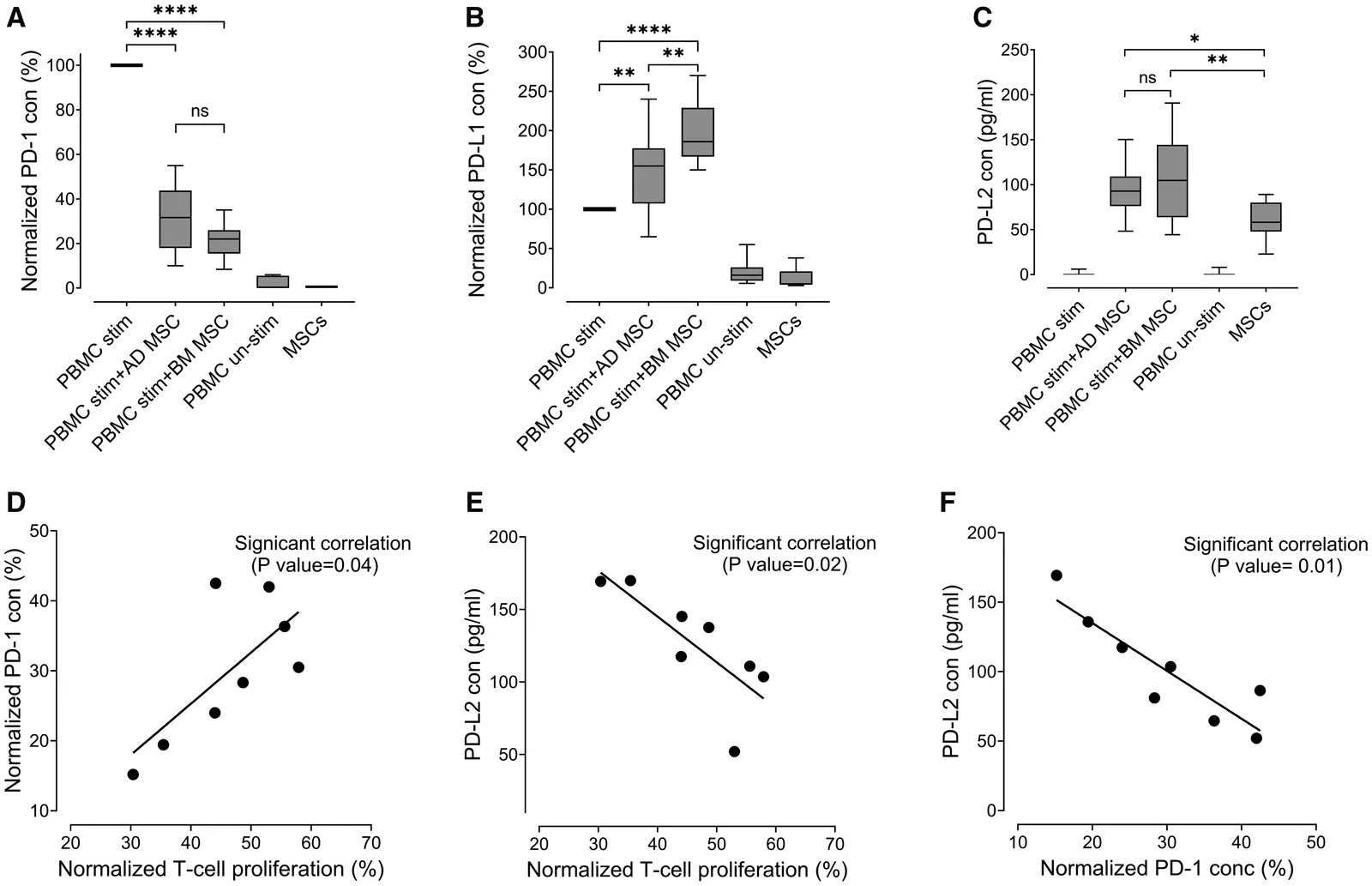
PD-1 axis protein levels and their correlation with T-cell proliferation in MLR. (A-C) concentration of soluble (A) PD-1, (B) PD-L1, and (C) PD-L2 in MLR culture supernatants. Soluble PD-1 and PD-L1 concentrations are normalized to the stimulated PBMC control, whereas PD-L2 is presented as an absolute concentration (pg/mL). Data are presented as median and interquartile ranges from four different experiments, including two sets of four biological replicates (four AD- and four BM-derived MSC donor preparations). Each biological replicate was analyzed in technical triplicate, and the mean of the technical replicates was used for analysis. Statistical significance was determined using one-way ANOVA followed by Tukey’s test. (*P ≤ .05, **P ≤ .01, ****P ≤ .0001). (D) Correlation between normalized soluble PD-1 levels and normalized T-cell proliferation (Spearman’s *ρ* = 0.70, *n* = 8, 95% CI, 0.03–0.94, *P* = .04). (E) Correlation between soluble PD-L2 levels and normalized T-cell proliferation (Spearman’s *ρ* = −0.70, *n* = 8, 95% CI, −0.95 to −0.17, *P* = .02). (F) Correlation between soluble PD-1 and soluble PD-L2 levels (Spearman’s *ρ* = −0.90, *n* = 8, 95% CI, −0.98 to −0.52, *P* = .01). Correlations were assessed using Spearman’s rank correlation analysis. AD, adipose; BM, bone marrow; MSC, mesenchymal stromal cells; MLR, mixed lymphocyte reaction; PD-1, programmed cell death protein 1; PD-L1, programmed death-ligand 1; PD-L2, programmed death-ligand 2; PBMC, peripheral blood mononuclear cells.

Moreover, soluble PD-L1 levels increased following PBMC activation and were further elevated when MSCs were present in co-culture, suggesting that both immune cells and MSCs contribute to PD-L1 production in this system (Figure 6B). We observed a distinct pattern for the PD-L2 secretion. Unlike PD-L1, soluble PD-L2 was not detected in activated PBMC cultures alone but was readily measurable when MSCs were present. Co-cultures containing BM- and AD-derived MSC showed measurable soluble PD-L2 (Figure 6C). We found that the concentration of PD-L2 inversely correlated with both the soluble PD-1 concentration and of T-cell proliferation. The strong inverse correlation between PD-L2 and the soluble PD-1 suggests coordinated regulation of the PD-1 signaling axis during MSCs-mediated immunosuppression (Figure 6D-F). However, no meaningful correlation was observed between PD-L1 levels and T-cell proliferation (Figure S2A) or between PD-L1 and PD-1 levels (Figure S2B).

Taking together, these findings indicate that MSCs-induced immune suppression in this system is associated with modulation of the PD-1 pathway and particularly with MSC-derived PD-L2 production, identifying a PD-1/PD-L2 signature associated with MSC functional potency.

## Discussion

In this study, we established a standardized immunosuppression-based assay framework for assessing MSC potency with improved robustness and mechanistic resolution. By systematically evaluating donor variability, culture conditions, and MSCs source, we demonstrated that assay configuration critically shapes functional readouts. Importantly, we identified reproducible patterns of immunosuppression and HLA-DR modulation on activated T-cells as a sensitive functional correlate, and link MSCs-mediated suppression to a PD-1/PD-L2-associated regulatory signature.

A key finding is that the dynamic range of allogeneic immune activation determines assay sensitivity. Restricting PBMC combinations to those exceeding 60% proliferation provided a reproducible high-activation state intended to model robust allogeneic immune responses, relevant to conditions such as acute GVHD. This threshold was selected empirically to ensure reproducible and sufficiently strong T-cell activation rather than representing a disease-specific quantitative marker. These responder conditions provide a standardized model of strong alloimmune activation for evaluating MSC-mediated immunosuppression. However, this approach may limit assay applicability to lower inflammatory settings, and future studies using donor combinations with a broader range of baseline proliferation will be valuable to further define assay performance across different immune activation states.^17^

Furthermore, MSCs were co-cultured with PBMCs at three different ratios. Among these, the 1:4 MSC: PBMC ratio was selected because it provided the greatest suppressive response and sufficient assay sensitivity to discriminate against MSC-mediated effects. Although this ratio exceeds expected *in vivo* MSC exposure, potency assays are designed to maximize analytical sensitivity rather than reproduce physiological cell ratios.^18^

Recent efforts toward assay harmonization emphasize the need for validated, reproducible potency platforms aligned with regulatory expectations, supporting the framework proposed here.^19^ Within this system, both BM- and AD-derived MSCs exert potent immunosuppressive effects, but with distinct functional profiles. While AD-derived MSCs demonstrated consistently higher suppression of T-cell proliferation, BM-derived MSCs tended to exert stronger suppression of late-stage T-cell activation, as evidenced greater HLA-DR downregulation. These findings suggest that tissue origin may influence specific aspects of MSC immunoregulatory function rather than uniformly altering the overall immunosuppressive capacity.^20^

We also observed source-dependent differences in CD142 and Podoplanin expression. Although these markers were not directly associated with immunosuppressive potency in the present study, they likely reflect intrinsic biological differences between BM- and AD-derived MSCs. Future studies should determine whether these phenotypic differences contribute to the distinct immunomodulatory profiles observed between MSC sources.^21^^,^^22^

Despite these differences, we observed a conserved, similar lower range of immunosuppression activity across MSC sources. When normalized, both BM- and AD-derived MSCs exhibit a remarkably similar proportional inhibitory activity. This observation suggests the presence of a shared lower functional range within the conditions of our assay, although the mechanisms underlying this apparent floor effect remain to be determined. This supports the concept that MSCs potency operates within defined biological limits rather than scaling indefinitely with dose. Consistent with this concept, prior studies have shown that MSCs-mediated inhibition of T-cell proliferation reaches a plateau governed by inducible suppressive pathways such as nitric oxide or prostaglandin signaling, rather than indefinitely scaling with cell dose.^23^

Importantly, our approach extends existing potency frameworks by defining a quantitative functional threshold within the MLR, enabling reproducible benchmarking of MSCs quality rather than relative donor ranking alone. This is in line with emerging concepts that MSCs potency is context-dependent and multidimensional. For example, Ren et al.,^24^ showed that IL-10 induction in THP-1 cells correlates with MSCs immunosuppressive activity, where higher IL-10 responses were associated with stronger inhibition of T-cell proliferation. However, their approach relied on ranking donors by relative performance. Thus, rather than only identifying “more potent” versus “less potent” MSCs, our assay establishes a reproducible functional threshold that may facilitate more consistent classification of MSCs potency and comparability across batches. This is also conceptually consistent with Wang et al.^25^ who demonstrated that cytokine licensing differentially enhances MSCs immunosuppressive pathways, and with Faircloth et al.,^26^ who showed that distinct functional outputs (angiogenesis vs immunosuppression) represent independent potency axes. Together, these studies reinforce the need for context-specific potency definitions, such as the immunosuppressive threshold established here.

Having established the functional framework for assessing MSC potency, we next examined whether specific immunomodulatory markers were associated with the observed suppressive activity. In contrast to some earlier reports, we did not observe a correlation between PD-L1 levels and T-cell proliferation.^27^ This difference likely reflects variations in assay design. In studies such as Guan et al., MSCs were pre-conditioned with IFN-γ, which promotes PD-L1 expression and links it to immunosuppressive activity. In our system, immune activation occurs without exogenous cytokine or pharmacological licensing, creating a more variable cytokine environment in which PD-L1 may not represent the predominant correlate of suppression. In addition, soluble PD-L1 may reflect contributions from multiple cell types rather than MSCs alone. By comparison, PD-L2 showed a stronger association with T-cell suppression, suggesting that it may represent a more informative marker of MSCs-mediated immunoregulation in this setting.

Although membrane-bound PD-1 pathway molecules mediate direct cell-cell checkpoint signaling, our preliminary flow cytometric analyses showed only modest surface expression changes that did not reliably distinguish experimental conditions. Consequently, surface marker expression, including MFI, did not provide a robust basis for distinguishing the experimental conditions. In contrast, soluble PD-1 pathway components provided reproducible quantitative measurements associated with MSC-mediated immunosuppression. Their ELISA-based quantification is objective, standardized, and compatible with quality control workflows, supporting their potential application in MSC potency assays.

Importantly, this distinction is further supported by differences in receptor binding properties between PD-L1 and PD-L2. PD-L2 binds PD-1 with higher affinity, a difference that may contribute to distinct checkpoint signaling properties.^28^ This higher affinity could contribute to enhanced PD-1 engagement in settings of active T-cell proliferation, where PD-1 expression is upregulated on activated T-cells. In this context, PD-L2-mediated engagement of PD-1 may more effectively suppress proliferative responses compared to PD-L1, particularly when ligand levels are limited. Our finding that PD-L2 levels inversely correlate with T-cell proliferation supports a model in which high-affinity PD-L2/PD-1 interactions preferentially regulate highly activated, PD-1 expressing T-cells within the MLR system.

Taken together, these findings suggest that PD-L2 is not redundant with PD-L1 but may represent a more dominant role ligand in regulating T-cell responses in allogeneic immune environments. This reinforces the concept that checkpoint biology in MSC-mediated immunoregulation is context-dependent and supports further evaluation of PD-L2 as a parameter in multi-dimensional potency assessment and as a potential candidate for incorporation into future release criteria.

A key conceptual advance of this study is the identification of HLA-DR downregulation on activated T-cells as a sensitive and mechanistically informative surrogate marker of MSCs activity. This dimension of T-cell activation is often overlooked in MSCs studies, where CD3/CD28-driven assays dominate early activation events such as CD25, thereby underestimating the relevance of late activation markers such as HLA-DR.^29^ The consistent and pronounced reduction of HLA-DR, particularly with BM-derived MSCs, suggests that MSCs not only limit T-cell expansion but also reshape activation state. Interestingly, exposure of MSCs to inflammatory environments has been shown to modulate their own HLA-DR expression, highlighting bidirectional crosstalk between MSCs and immune cells. Our findings extend this paradigm to T-cell HLA-DR modulation, revealing a previously underappreciated layer of MSCs-mediated immune regulation.

At the subset level, MSCs preferentially suppressed CD8^+^ T-cell proliferation, restoring the CD4/CD8 ratios toward baseline. This suggests selective control of cytotoxic T-cell responses, consistent with other studies showing that pathways such as IDO can specifically inhibit CD8^+^ T-cells. This selective effect is consistent with prior work implicating metabolic pathways such as IDO in targeting cytotoxic T-cell responses.^30^ Our data suggests that such mechanisms may act in concert with PD-1/PD-L2 signaling to restrain CD8^+^ T-cell expansion, particularly under inflammatory conditions.

Mechanistically, our findings identify the PD-1/PD-L2 axis as a candidate pathway associated with MSCs-mediated immunoregulation. Increased PD-L2 alongside reduced soluble PD-1, and its inverse correlation with T-cell proliferation, support a direct role for MSCs-derived PD-L2 in suppressing T-cell activation.^31^^,^^32^ Notably, our findings extend prior work by Davies et al. who demonstrated MSCs secretion of PD-1 ligands under CD3/CD28 stimulation conditions. In contrast, our MLR model captures antigen-driven activation, identifying PD-L2 as a potential context-dependent potency marker under these conditions.^14^

Taken together, these results support a multi-layered model of MSCs function integrating proliferation control, modulation of activation states, subset-specific effects, and checkpoint signaling. By defining quantifiable and mechanistically linked parameters, this work advances the development of standardized potency assays for MSC-based therapies.

Several limitations should be acknowledged. While the MLR provides a robust and controlled platform to interrogate MSCs-mediated immunomodulation, it remains an inherently reductionist *in vitro* system that cannot fully capture the complexity, spatial organization, and dynamic regulation of immune responses *in vivo*. Although functional assays are widely required by regulatory authorities for the release of MSCs-based products, a consistent and predictive correlation between *in vitro* potency readouts and clinical outcomes has yet to be established. Nonetheless, such assays are essential for ensuring functional consistency under controlled conditions. Additionally, the use of soluble checkpoint measurements does not reflect cell-bound dynamics or spatial organization. Future studies integrating single-cell transcriptomics, spatial profiling, and *in vivo* validation will be essential to further refine these mechanistic insights.

In conclusion, this work provides a standardized and mechanistically anchored framework for assessing MSCs immunomodulatory potency. Identification of HLA-DR modulation, preferential CD8^+^ T-cell suppression, and PD-L2 associated signaling as key functional correlates offers new tools for both research and clinical translation. These findings contribute to a more precise definition of MSC activity and support the development of next-generation potency assays designed to improve functional characterization and comparability of MSC products.

## Supplementary Material

szag090_Supplementary_Data

## Data Availability

All data generated or analysed during this study are included in this published article and its supplementary information files. Additional raw data are available from the corresponding author upon reasonable request.

## References

[1] Dominici M , Le Blanc K, Mueller I, et al Minimal criteria for defining multipotent mesenchymal stromal cells: the international society for cellular therapy position statement. Cytotherapy. 2006;8:315-317. 10.1080/14653240600855905 16923606

[2] Kou M , Huang L, Yang J, et al Mesenchymal stem cell-derived extracellular vesicles for immunomodulation and regeneration: a next generation therapeutic tool. Cell Death Dis. 2022;13:580. 10.1038/s41419-022-05034-x PMC9252569 35787632

[3] Vizoso FJ , Eiro N, Cid S, et al Mesenchymal stem cell secretome: toward cell-free therapeutic strategies. Int J Mol Sci. 2017;18:1852. 10.3390/ijms18091852 PMC5618501 28841158

[4] Yang G , Fan X, Liu Y, et al Immunomodulatory mechanisms and therapeutic potential of mesenchymal stem cells. Stem Cell Rev Rep. 2023;19:1214-1231. 10.1007/s12015-023-10539-9 PMC10103048 37058201

[5] Boyman O , Sprent J. The role of interleukin-2 during homeostasis and activation of the immune system. Nat Rev Immunol. 2012;12:180-190. 10.1038/nri3156 22343569

[6] Kumar BV , Connors TJ, Farber DL. Human T cell development, localization, and function throughout life. Immunity. 2018;48:202-213. 10.1038/nri3156 PMC5826622 29466753

[7] Zaunders JJ , Munier ML, Seddiki N, et al High levels of human antigen-specific CD4+ T cells in peripheral blood revealed by stimulated coexpression of CD25 and CD134 (OX40). J Immunol. 2009;183:2827-2836. 10.4049/jimmunol.0803548 19635903

[8] Le Blanc K , Frassoni F, Ball L, et al; Developmental Committee of the European Group for Blood and Marrow Transplantation. Mesenchymal stem cells for treatment of steroid-resistant, severe, acute graft-versus-host disease: a phase II study. Lancet. 2008;371:1579-1586. 10.1016/S0140-6736(08)60690-X 18468541

[9] European Medicines Agency. *Guidelines on Human Cell-Based Medicinal Products*. European Medicines Agency; 2020.

[10] Sawada R , Kusakawa S, Kusuhara M, et al Increasing robustness of in vitro assay for immunosuppressive effect of mesenchymal stromal/stem cells: the role of inflammatory cytokine production by peripheral blood mononuclear cells. Regen Ther. 2025;28:321-332. 10.1016/j.reth.2024.12.016 PMC11773150 39877252

[11] Ouzin M , Kogler G. Mesenchymal stromal cells: heterogeneity and therapeutical applications. Cells. 2023;12:2039. 10.3390/cells12162039 PMC10453316 37626848

[12] Li X , Bai J, Ji X, et al Comprehensive characterization of four different populations of human mesenchymal stem cells as regards their immune properties, proliferation and differentiation. Int J Mol Med. 2014;34:695-704. 10.3892/ijmm.2014.1821 PMC4121354 24970492

[13] Sharpe AH , Pauken KE. The diverse functions of the PD-1 inhibitory pathway. Nat Rev Immunol. 2018;18:153-167. 10.1038/nri.2017.108 28990585

[14] Davies LC , Heldring N, Kadri N, et al Mesenchymal stromal cell secretion of programmed death-1 ligands regulates T cell mediated immunity. Stem Cells. 2017;35:766-776. 10.1002/stem.2509 PMC5599995 27671847

[15] Aghbash P , Mehdizadeh F, Pourbeiragh G, et al PD-L1 and PD-1 in immune regulation and their implications in blood cancers. Adv Cancer Biol Metastasis. 2024;11:100125.

[16] Samarasinghe S , Mancao C, Pule M, et al Functional characterization of alloreactive T cells identifies CD25 and CD71 as optimal targets for a clinically applicable allodepletion strategy. Blood. 2010;115:396-407. 10.1182/blood-2009-08-235895 19890093

[17] Le Blanc K , Tammik L, Sundberg B, et al Mesenchymal stem cells inhibit and stimulate mixed lymphocyte cultures and mitogenic responses independently of the major histocompatibility complex. Scand J Immunol. 2003;57:11-20. 10.1046/j.1365-3083.2003.01176.x 12542793

[18] Galipeau J , Krampera M. The challenge of defining mesenchymal stromal cell potency assays and their potential use as release criteria. Cytotherapy. 2015;17:125-127. 10.1016/j.jcyt.2014.12.008 25593076

[19] Nicotra T , Desnos A, Halimi J, et al Mesenchymal stem/stromal cell quality control: validation of mixed lymphocyte reaction assay using flow cytometry according to ICH Q2(R1). Stem Cell Res Ther. 2020;11:426. 10.1186/s13287-020-01947-6 PMC7531151 33004063

[20] Haddad R , Saldanha-Araujo F. Mechanisms of T-cell immunosuppression by mesenchymal stromal cells: what do we know so far? Biomed Res Int. 2014;2014:216806. 10.1155/2014/216806 PMC4082893 25025040

[21] Hoang VT , Le DS, Hoang DM, et al Impact of tissue factor expression and administration routes on thrombosis development induced by mesenchymal stem/stromal cell infusions: re-evaluating the dogma. Stem Cell Res Ther. 2024;15:56. 10.1186/s13287-023-03582-3 PMC10900728 38414067

[22] Ward LSC , Sheriff L, Marshall JL, et al Podoplanin regulates the migration of mesenchymal stromal cells and their interaction with platelets. J Cell Sci. 2019;132:jcs222067. 10.1242/jcs.222067 PMC6432720 30745334

[23] Zinöker S , Vage YT. Rat mesenchymal stromal cells inhibit T cell proliferation but not cytokine production through inducible nitric oxide synthase. Front Immunol. 2012;3:62. 10.3389/fimmu.2012.00062 PMC3341954 22566943

[24] Ren J , Szombath G, Vitale-Cross L, et al The potential use of THP-1, a monocytic leukemia cell line, to predict immune-suppressive potency of human bone-marrow stromal cells (BMSCs) in vitro: a pilot study. Int J Mol Sci. 2023;24:13258. 10.3390/ijms241713258 PMC10488111 37686058

[25] Wang J , Zhou Y, Donohoe E, et al Immunomodulatory potential of cytokine-licensed human bone marrow-derived mesenchymal stromal cells correlates with potency marker expression profile. Stem Cells. 2024;42:1040-1054. 10.1093/stmcls/sxae053 PMC11630899 39208292

[26] Faircloth TU , Temple S, Parr RN, et al Vascular endothelial growth factor secretion and immunosuppression are distinct potency mechanisms of human bone marrow mesenchymal stromal cells. Stem Cells. 2024;42:736-751. 10.1093/stmcls/sxae040 38826008

[27] Guan Q , Li Y, Shpiruk T, et al Inducible indoleamine 2,3-dioxygenase 1 and programmed death ligand 1 expression as potency markers for mesenchymal stromal cells. Cytotherapy. 2018;20:639-649. 10.1016/j.jcyt.2018.02.003 29548707

[28] Latchman Y , Wood CR, Chernova T, et al PD-L2 is a second ligand for PD-1 and inhibits T cell activation. Nat Immunol. 2001;2:261-268. 10.1038/85330 11224527

[29] Zhang Y , Pilati D, Dong M, et al The interaction of PD-1/PD-L1 and its inhibition by anti-PD-L1 antibody at the single-molecule level. FEBS Lett. 2026;600:1237-1247. 10.1002/1873-3468.70245 41388338

[30] Hong J , Hueckelhoven A, Wang L, et al Indoleamine 2,3-dioxygenase mediates inhibition of virus-specific CD8+ T cell proliferation by human mesenchymal stromal cells. Cytotherapy. 2016;18:621-629. 10.1016/j.jcyt.2016.01.009 27008974

[31] Parvez A , Choudhary F, Mudgal P, et al PD-1 and PD-L1: architects of immune symphony and immunotherapy breakthroughs in cancer treatment. Front Immunol. 2023;14:1296341. 10.3389/fimmu.2023.1296341 PMC10722272 38106415

[32] Yearley JH , Gibson C, Yu N, et al PD-L2 expression in human tumors: relevance to anti-PD-1 therapy in cancer. Clin Cancer Res. 2017;23:3158-3167. 10.1158/1078-0432.CCR-16-1761 28619999

