# Supplementary material for: HLA-DR modulation and PD-1/PD-L2 checkpoint signaling identify a mechanistic potency axis for mesenchymal stromal cell immunosuppression
Source: Stem Cells Transl Med. 2026 Nov 5;15(11):szag090. doi: 10.1093/stcltm/szag090 (PMC13738804; doi:10.1093/stcltm/szag090)
Supplement: Supplementary file 1 [file szag090_supplementary_data.docx]

**Supplementary information**

**HLA-DR MODULATION AND PD-1/PD-L2 CHECKPOINT SIGNALLING IDENTIFY A MECHANISTIC POTENCY AXIS FOR MESENCHYMAL STROMAL CELL IMMUNOSUPPRESSION**

**Running title:** HLA-DR and PD-1/PD-L2 Axis Define MSC Potency

Mahin Nikougoftar Zarif^1^, Katia Lefsihane^1^, Neda Khanlarkhani^1^, Lina Sorvik^1^, Jan Fredrik Talts^1^, Katarina Le Blanc^2^, Nadir Kadri^2^*

1: Cellcolabs AB, Stockholm, Sweden

2: Department of Laboratory medicine, Karolinska Institute, Huddinge, Sweden

**Author contributions**

Author 1: Conception and design, Provision of study material or patients, Collection of data, Data analysis and interpretation, Manuscript writing, Final approval of manuscript

Author 2: Manuscript review, Collection of data

Author 3: Manuscript review, Data analysis and interpretation

Author 4: Manuscript review, Provision of study material or patients

Author 5: Manuscript review, Financial support, Final approval of manuscript

Author 6: Conception and design, Provision of study material or patients, Manuscript review

Author 7: Conception and design, Provision of study material or patients, Financial support, Data analysis and interpretation, Manuscript writing, Final approval of manuscript

*: Corresponding author: Nadir Kadri, Assistant professor, Department of Laboratory medicine, Karolinska Institute, Alfred Nobels alle 8, Huddinge, Sweden, PO. Box:14152, Tel: +46707191494, [nadir.kadri@ki.se](mailto:nadir.kadri@ki.se)

Key words: Mesenchymal stromal cells, HLA-DR, PD-1, Potency assay, Mixed lymphocyte reaction

**Supplementary Table. PBMC and MSC samples used in this study.**
(A) Combinations of PBMCs from different donors used to assess variability in T-cell proliferation. PBMC donors ranged in age from 25 to 52 years. (B and C) MSC samples, including their tissue sources and batch information, used in the experiments. All four AD MSC samples and two BM-derived MSC samples were obtained from female donors, whereas the remaining two BM-derived MSC samples were obtained from male donors. MSC donors ranged in age from 21 to 46 years.

PBMC, peripheral blood mononuclear cells; MSC, mesenchymal stromal cells; AD, adipose; BM, bone marrow.

**Supplementary Figure 1. Representative analysis of T-cell proliferation across different ratios and controls.**

(A) Representative flow cytometry gating strategy for identification of viable CD3⁺ T cells. Cells were sequentially gated based on lymphocyte characteristics (FSC-A/SSC-A), single, viable cells, and CD3⁺ cells. The CD3⁺ population was subsequently used for assessment of T-cell proliferation by CellTrace Violet (CTV) dilution. (B) Representative CellTrace Violet (CTV) profiles showing T-cell proliferation. The distinct peak on the right represents non-proliferating T cells, whereas successive peaks with decreasing fluorescence intensity, corresponding to leftward shifts, represent successive proliferating T-cell generations. T-cell proliferation was quantified based on CTV dye dilution. (C) NTPR (%) at MSC ratios of 1:4, 1:8, and 1:16 for AD and BM-derived MSCs. Data is presented as mean ± SD from eight biological replicates (four AD and four BM-derived MSC donor preparations), with each biological replicate analysed in technical triplicate.

CTV, CellTrace Violet; NTPR, normalized T-cell proliferation reduction; MSC, mesenchymal stromal cells; PBMC, peripheral blood mononuclear cells; AD, adipose; BM, bone marrow; MLR, mixed lymphocyte reaction; SD, standard deviation.

**Supplementary Figure 2. Soluble PD-L1 levels and their correlation with T-cell proliferation and soluble PD-1 levels in MLR.**

(A) Correlation between soluble PD-L1 levels and T-cell proliferation (ρ = −0.10, n = 8, 95% CI = −0.75 to 0.65, P = 0.81). (B) Correlation between soluble PD-L1 levels and PD-1 levels (ρ = −0.50, n = 8, 95% CI = −0.88 to 0.35, P = 0.16). Correlations were assessed using Spearman's rank correlation analysis. Data was obtained from eight biological replicates (four AD and four BM-derived MSC donor preparations).

PD-L1, programmed death-ligand 1; PD-1, programmed cell death protein 1; MLR, mixed lymphocyte reaction.
